# Editorial: Identifying novel biomarkers in bladder cancer

**DOI:** 10.3389/fonc.2023.1191736

**Published:** 2023-07-04

**Authors:** Thorsten H. Ecke, Yair Lotan, Thierry Massfelder

**Affiliations:** ^1^ Department of Urology, Helios Hospital Bad, Saarow, Germany; ^2^ Deparment of Urology, Universitätsmedizin Berlin Charité, Berlin, Germany; ^3^ Department of Urology, University of Texas Southwestern Medical Center, Dallas, TX, United States; ^4^ INSERM (French National Institute of Health and Medical Research) UMR_S1260, Université de Strasbourg, Regenerative Nanomedicine, Centre de Recherche en Biomédecine de Strasbourg, Strasbourg, France

**Keywords:** bladder cancer (BCa), urology, tumor marker, urine, diagnostics

Urothelial carcinoma (UC) of the bladder is the tenth most diagnosed cancer worldwide and represents a significant cause of morbidity and mortality (1). UC is a heterogeneous disease with significant differences in management and prognosis for non-muscle invasive and muscle invasive stages (2). The increased knowledge regarding molecular changes associated with development and growth of bladder cancer has accelerated research into the disease (3). Methodological developments have contributed to a better understanding of the biology and heterogeneity of UC. This new understanding has also led to the development of new biomarkers and approaches for targeted therapy (4).

Cystoscopy and cytology are still mentioned as gold standard for the detection and surveillance of bladder cancer with unequal combined overall sensitivity and specificity. However, they have only limited sensitivity in detecting small lesions of the urinary tract and flat lesions such as carcinoma *in situ* that are thus particularly challenging. For such cases urine cytology is still the most used non-invasive test detecting bladder cancer during follow-up. Despite its high specificity of approximately 86%, the limitation lies in low sensitivity of only 50% (5), especially for low-grade tumors (6, 7). With the absence of reliable cost-effective urinary biomarkers, the confirmation of suspected carcinomas of the urinary tract and the subsequent life-long surveillance for relapse is nowadays still undertaken by cystoscopically examinations, which represents significant costs for healthcare systems (8).

There is a wide range of potential clinical uses for markers. Molecular markers could improve detection of disease in hematuria patients, improve surveillance for NMIBC, detect residual disease after therapy, predict response to treatment, select patients for more aggressive treatment and identify patients for targeted therapies. Markers can evaluate urine, serum, plasma and can identify alterations in proteins, DNA, RNA, epigenetic changes, cellular changes and be individual or panels. Most importantly, markers need validation and evaluation of clinical benefit.

Studies comparing different rapid tests to the gold standard, urine cytology based on the Paris system for reporting (9) and integrating clinical parameters such as haematuria, ECOG performance score, smoking behavior and others (10) are needed (11).

An incomplete understanding of tumor biology and responsiveness to current therapy strategies has led to numerous studies focusing on differentiating molecular subtypes of bladder cancer (12). Basal and luminal carcinomas have been described as the main subtypes for NMIBC and MIBC, along with tumor heterogeneity at genetic and molecular levels. Similarities exist to breast cancer subtypes, for which targeted therapies are well established (13, 14). Protein expressions of markers for basal (CK5/6, CK14, CD 40) and luminal subtypes (CK20, GATA3, ERß, Uroplakin II, HER2/neu, FGFR3) have been found. Furthermore, sub-classifications have been described and appear promising for targeted therapies as they have shown distinct differences in biological behavior and chemotherapy sensitivity (15).

For example, erdafitinib has been approved for the treatment of advanced and metastatic bladder cancer patients bearing mutations or fusions of FGFR3 (16). However, the combination of dual tyrosine kinase inhibitors targeting FGFR3 and ERBB2 has not yet investigated. Given the endogenous interplay and correlations of both pathways, this combination could provide synergistic effects, and overcome limited activity of single agent treatment.

There is a great need for further investigations of molecular markers for bladder cancer in the context of risk stratification and for developing combined targeted therapy options to prevent progression and cancer-related death. According to newer publications, FGFR3 mutation analysis study on bladder cancer patients should be performed for all stages and grades (12). There are ongoing studies of FGFR inhibitors in patients who received Bacillus Calmette-Guérin (BCG) and recurred with high-risk non-muscle-invasive bladder cancer (NMIBC) including those with intermediate risk disease (ClinicalTrials.gov Identifier: NCT04172675).

It is mandatory perceiving that marker systems are playing an important role in all fields of bladder cancer: as an alternative or additional tool for cystoscopy during follow-up, as predictor and prognostic instrument during decisions for systemic therapies or as screening tool for detecting bladder cancer in high-risk groups.

This Research Topic has the main aim of offering possibilities to publish new research results in the basic and translational research field of bladder cancer. During the editorial process of this Research Topic we have appreciated that significant progress has been recently done in bladder cancer research and that efforts should be pursued by fostering extensive cooperation between the scientific and medical communities to translate evidence-based research into clinical practice. The identification and validation of bladder cancer markers for predicting recurrence and progression will contribute to establish better treatments for the individual patient based on its predicted response and their specific genetic and molecular characteristics, and molecular staging will allow selection of tumors that will require systemic treatment.

The studies in this Research Topic cover the major areas of developing interest in bladder cancer research.

There were several novel markers evaluated in this Research Topic. Wang et al. studied ferroptosis regulators and identified GCLM as a tumor promotor and immunological biomarker in bladder cancer. Feng et al. evaluated the prognostic significance basement membrane-associated lncRNA in Bladder Cancer.

There were several articles evaluating new diagnostic markers for bladder cancer including a study by Lee et al. evaluating alpha-2-macroglobulin in urinary extracellular vesicles and a study by Bian et al. evaluating urinary exosomal long non-coding RNAs as noninvasive biomarkers for diagnosis of bladder cancer. Su et al. analyzed exosome-derived long non-coding RNAs as non-invasive biomarkers of bladder cancer.

To improve imaging of disease, Hao et al. study near-infrared targeted imaging using ICG-anti-CD47.

There were several studies evaluating bladder cancer prognosis. Wang et al. review the impact of cuproptosis-related genes on bladder cancer prognosis, tumor microenvironment invasion, and drug sensitivity. Castaneda et al. identify Novel Biomarkers associated with Bladder Cancer Treatment Outcomes. Wang et al. use proteomics analyses to identify CLIC1 as a predictive biomarker for bladder cancer staging and prognosis. Li et al. study endothelial-related molecular subtypes for bladder cancer patients, and Xiong et al. study inflammation-related lncRNAs in bladder cancer. You et al. study novel pyroptosis-related gene signatures and Xiao et al. evaluate impact of fatty acid metabolism, inflammation and hypoxia on bladder cancer.

Several studies developed prediction models for bladder cancer. Chen et al. study the tumor microenvironment to develop a nomogram to predict lymph node metastases. Bieri et al. use a modified Immunoscore to improve prediction of progression-free survival in patients with non-muscle-invasive bladder cancer. Luo et al. develop a novel prognostic model based on cellular senescence-related gene signatures for bladder cancer

A study by Song et al. evaluated aliphatic acid metabolism in bladder cancer with the goal of guiding therapeutic treatment. A study by Wang et al. studied the mechanism by which RAC3 inhibition induces autophagy to impair metastasis in bladder cancer cells via the PI3K/AKT/mTOR pathway.

All editors of this Research Topic thank all submitting authors for their work. The lead editors would like to thank all editors and reviewers for the time spent in assigning reviews, commenting on submitted manuscripts as well as reviewing. As editorial team, we hope that this special issue will prove useful in planning bladder cancer research in next future.

## Author contributions

All authors listed have made a substantial, direct, and intellectual contribution to the work and approved it for publication.
